# Ruxolitinib as an alternative to hydroxyurea in essential thrombocythemia in the setting of nonhealing ulcers

**DOI:** 10.1016/j.jdcr.2026.04.030

**Published:** 2026-04-24

**Authors:** Chloe Ricke, Frank Ross

**Affiliations:** a NYU Grossman School of Medicine, New York, New York; b Department of Surgery, NYU Grossman School of Medicine, New York, New York

**Keywords:** drug-induced ulcers, essential thrombocythemia, hydroxyurea, nonhealing ulcers, ruxolitinib, wound healing

## Introduction

Hydroxyurea (HU) is a cytotoxic antimetabolite effective in treating myeloproliferative disorders. The cytotoxic abilities of HU have been theorized to contribute to its cutaneous side effects, such as hyperpigmentation, xerosis, skin atrophy, alopecia, oral stomatitis, and cutaneous ulcers.^1^ Despite being the first-line therapy for essential thrombocythemia (ET), 20% of patients become resistant or intolerant to HU therapy. The JAK1/JAK2 inhibitor ruxolitinib has been shown to be a safe and effective alternative treatment for myeloproliferative disorders in patients who are resistant or intolerant to HU.^2^ We report a case of essential thrombocythemia complicated by chronic, nonhealing ankle ulcers during HU therapy that improved after discontinuation of HU and transition to ruxolitinib.

## Case Report

A 69-year-old man was diagnosed with essential thrombocythemia (ET) in 2016 after routine testing revealed marked thrombocytosis (platelets 1244 × 10^9^/L). Bone marrow biopsy confirmed ET with a CALR mutation, and HU 500 mg 3 times daily was initiated. His platelet counts remained well controlled; however, in 2019 he developed a painful ulcer over the left medial malleolus. Arterial studies confirmed intact perfusion, and the ulcer healed with antibiotics, Unna boot compression, and local wound care.

In 2020, new ulcers developed over the medial and lateral malleoli of the same ankle, which recurred despite compression therapy and antibiotics. Vascular evaluation revealed May–Thurner syndrome, and a left iliac vein stent was placed with partial improvement. HU was gradually reduced as platelet counts normalized, and by late 2021, the ulcers had healed temporarily.

In early 2024, following hammer-toe surgery that prevented use of compression stockings, both malleolar ulcers reopened. Duplex studies demonstrated venous reflux, and the left great saphenous vein was ablated. Despite debridement, compression therapy, and local wound care, the ulcers remained nonhealing. Angiography in early 2025 revealed tibial artery calcification and focal popliteal stenosis, which was treated with atherectomy and angioplasty, but the left lateral ulcer persisted despite these interventions.

**Fig 1:**
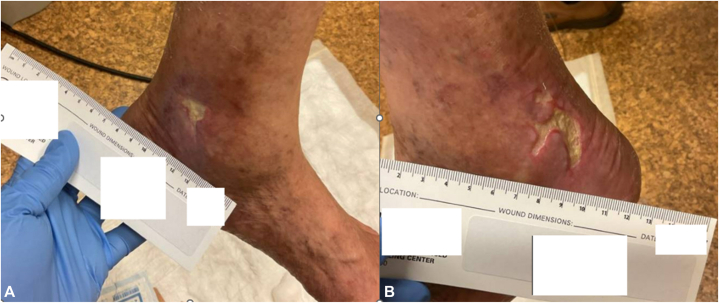
**A,** Left medial ankle ulcer and **(B)** left lateral ankle ulcer at initial presentation, approximately 5 y after initiation of hydroxyurea.

**Fig 2:**
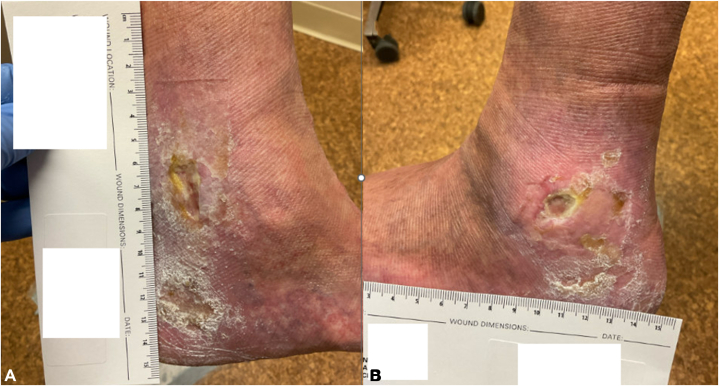
**A,** Left medial ankle ulcer and **(B)** left lateral ankle ulcer approximately 8 y after hydroxyurea initiation, demonstrating progression despite ongoing wound care.

**Fig 3:**
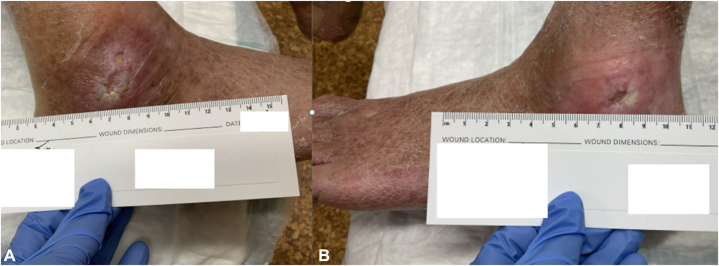
**A,** Left medial ankle ulcer and **(B)** left lateral ankle ulcer prior to transition to ruxolitinib, with persistent nonhealing ulcers despite standard wound care.

**Fig 4:**
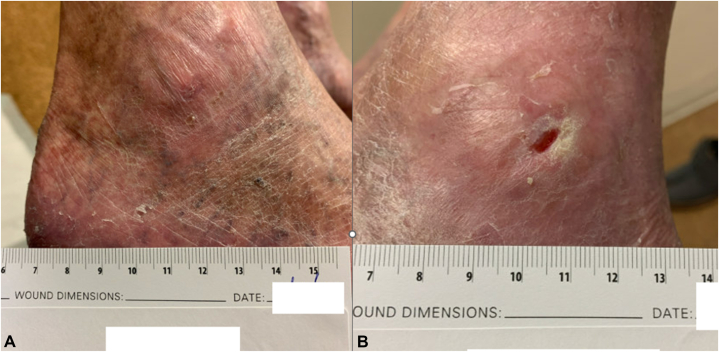
**A,** Left medial ankle ulcer and **(B)** left lateral ankle ulcer 4 m after discontinuation of hydroxyurea, demonstrating clinical with subsequent patient-reported closure of the previously persistent lateral ulcer.

In May 2025, while still on HU, the patient developed new ulcers on the right ankle, further supporting a medication-related etiology. Because of the chronicity of his left-sided ulcer and the new contralateral lesions despite adequate perfusion, HU was discontinued and ruxolitinib was initiated at a dose of 5 mg twice daily. Within 4 mo, all ulcers had clinically healed. The previously persistent left lateral ulcer, last measured at 0.7 × 0.2 × 0.3 cm, was subsequently reported by the patient to be fully closed. The patient’s platelet counts remained within the target range without adverse effects. Clinical progression and subsequent healing of the ulcers are shown in Fig 1, Fig 2, Fig 3 to 4, and laboratory trends are summarized in Table I.

**Table I:** Laboratory trends before and after transition from HU to ruxolitinib

Parameter	Reference range	Baseline (pre-HU)	On HU	Postswitch (ruxolitinib)
WBC (× 10^9^/L)	4.0-11.0	7.5	4.5	5.3
Hemoglobin (g/dL)	13.0-17.0	12.3	9.4	9.4
Hematocrit (%)	40-50	-	26.6	27.4
Platelets (× 10^9^/L)	150-450	1244	466	448
MCV (fL)	80-100	-	135.7	110.9
LDH (U/L)	125-243	-	190	246
Creatinine (mg/dL)	0.6-1.2	-	0.86	0.94
AST (U/L)	<40	-	38	47
ALT (U/L)	<41	-	29	37
JAK2 V617 F mutation	-	Negative	Negative	Negative
Medication dose	-	-	1000 mg/d	10 mg/d

*ALT,* Alanine aminotransferase; *AST,* aspartate aminotransferase; *HU,* hydroxyurea; *JAK,* Janus kinase; *LDH,* lactate dehydrogenase; *MCV,* mean corpuscular volume; *WBC,* white blood cell.

## Discussion

Despite its efficacy in treating myeloproliferative neoplasms, HU is associated with well-documented dermatologic side effects, which can render the medication intolerable for many patients. HU acts during the S phase of cellular replication, causing direct cytotoxic damage in tissues with high cell turnover through inhibition of DNA synthesis.^3^ HU-induced ulcers are typically perimalleolar, intensely painful, and often bilateral, with severity correlating with HU dosage. Minor trauma, such as shoe rubbing against the malleolus, can precipitate ulcer formation, as the cytostatic effects of HU impair basal epidermal regeneration.^4^ Biopsy demonstrates epidermal atrophy, dermal fibrosis, and scar tissue,^3^ while macrocytosis of vascular endothelial cells and reduced RBC adherence may impair microcirculatory blood flow, contributing to relative ischemia and delayed wound healing.^4^^,^^5^

Healing of persistent ulcers after HU discontinuation has been well documented. In a study of 41 patients who developed ulcers on HU therapy, 80% healed rapidly following withdrawal of treatment.^3^ A similar case report described a patient with polycythemia vera who developed severe, bilateral necrotic ulcers persisting for 5 y, which healed within 6 mo after switching from HU to ruxolitinib and undergoing skin grafting.^6^ The mechanism underlying this response remains unclear. Resolution of persistent ulcers after HU discontinuation has been well described, suggesting that removal of the cytotoxic insult may be the primary driver of ulcer resolution. Activation of the JAK/STAT3 pathway can inhibit apoptosis and induce antimicrobial molecules, regulating the proliferation and differentiation of cells central to wound healing.^7^ While JAK inhibitors have paradoxically been associated with ulcer development,^8^ ruxolitinib may not be directly cytotoxic, and removal of the primary insult (HU) may suffice to permit healing. However, a contributory role of ruxolitinib cannot be excluded, although hydroxyurea discontinuation is likely the primary driver of ulcer healing.

Clinically, several principles guide the management of nonhealing ulcers in patients on HU. First, a high index of suspicion for HU-related ulceration is essential, and a careful review of the patient’s medication list should always be performed. Second, comorbid conditions such as peripheral arterial disease and chronic venous insufficiency must be addressed concurrently, as they may exacerbate or mask HU-induced lesions. Third, although biopsy is rarely required, it may be warranted if the diagnosis is uncertain or there is concern for malignancy or vasculitis. Finally, a broad differential diagnosis, including venous, arterial, neuropathic, and vasculitic etiologies, should be maintained to ensure other causes of chronic ulceration are not overlooked.

This case highlights that in patients with ET who develop persistent, nonhealing ulcers, HU should be considered as a potential contributing factor, particularly when ulcers fail to respond to appropriate wound care, including debridement, compression therapy, topical therapy, and optimization of arterial and venous insufficiency. In such cases, after exclusion of other etiologies, discontinuation of hydroxyurea should be strongly considered. Transition to ruxolitinib can allow for continued hematologic control while ulcer healing occurs.

## Conflicts of interest

None disclosed.

## References

[1] Bulte C.A., Hoegler K.M., Kutlu Ö., Khachemoune A. (2021). Hydroxyurea: a reappraisal of its cutaneous side effects and their management. Int J Dermatol.

[2] Verstovsek S., Passamonti F., Rambaldi A. (2017). Ruxolitinib for essential thrombocythemia refractory to or intolerant of hydroxyurea: long-term phase 2 study results. Blood.

[3] Sirieix M., Debure C., Baudot N. (1999). Leg ulcers and hydroxyurea: forty-one cases. Arch Dermatol.

[4] Quattrone F., Dini V., Barbanera S., Zerbinati N., Romanelli M. (2013). Cutaneous ulcers associated with hydroxyurea therapy. J Tissue Viability.

[5] Adragna N.C., Fonseca P., Lauf P.K. (1994). Hydroxyurea affects cell morphology, cation transport, and red blood cell adhesion in cultured vascular endothelial cells. Blood.

[6] Shanmugam V.K., McNish S., Shara N. (2013). Chronic leg ulceration associated with polycythemia vera responding to ruxolitinib (Jakafi®). J Foot Ankle Surg.

[7] Song Q., Xie Y., Gou Q., Guo X., Yao Q., Gou X. (2017). JAK/STAT3 and Smad3 activities are required for the wound healing properties of *Periplaneta americana* extracts. Int J Mol Med.

[8] Aslan Candır B., Yiğenoğlu T.N., Kızıl Çakar M., Dal M.S., Altuntaş F. (2022). Skin ulcers caused by ruxolitinib in a patient with chronic cutaneous graft-versus-host disease. J Oncol Pharm Pract.

